# Supplementary material for: NMNAT promotes glioma growth through regulating post-translational modifications of P53 to inhibit apoptosis
Source: eLife. 2021 Dec 17;10:e70046. doi: 10.7554/eLife.70046 (PMC8683086; doi:10.7554/eLife.70046)
Supplement: Figure 3—source data 1. [file elife-70046-fig3-data1.doc]

List of siRNA sequence

| gene | Sense (5’-3’) | Antisense (3’-5’) |
| --- | --- | --- |
| NMNAT1-1 | GGAAACACCGGAGCAACAUTT | AUGUUGCUCCGGUGUUUCCTT |
| NMNAT1-2 | GGUCAUCAUGGCAGAACUUTT | AAGUUCUGCCAUGAUGACCTT |
| NMNAT2-1 | GCAUCCUCUCCAAUGUCAATT | UUGACAUUGGAGAGGAUGCTT |
| NMNAT2-2 | CCAUUUACCAGAACAGCAATT | UUGCUGUUCUGGUAAAUGGTT |
| Negative Control | UUCUCCGAACGUGUCACGUTT | ACGUGACACGUUCGGAGAATT |

List of NMNAT primer

| gene | Forward | Reverse |
| --- | --- | --- |
| NMNAT1 | ACAAAAGCTGTGCCAAAGGTC | TAGTTGGCCACGATTTGGGT |
| NMNAT2 | GCCGACACAGACCGAATCAT | GGCCAGCCTGCTCTTGG |
| β-actin | CACCCTGAAGTACCCCATGG | TGCCAGATTTTCTCCATGTCG |

**Figure 3-source data 1**

siRNA sequences for NMNAT1/2 knockdown and primer sequences for PCR.
