# Supplementary material for: NMNAT promotes glioma growth through regulating post-translational modifications of P53 to inhibit apoptosis
Source: eLife. 2021 Dec 17;10:e70046. doi: 10.7554/eLife.70046 (PMC8683086; doi:10.7554/eLife.70046)
Supplement: Figure 3—figure supplement 1—source data 1. [file elife-70046-fig3-figsupp1-data1.doc]

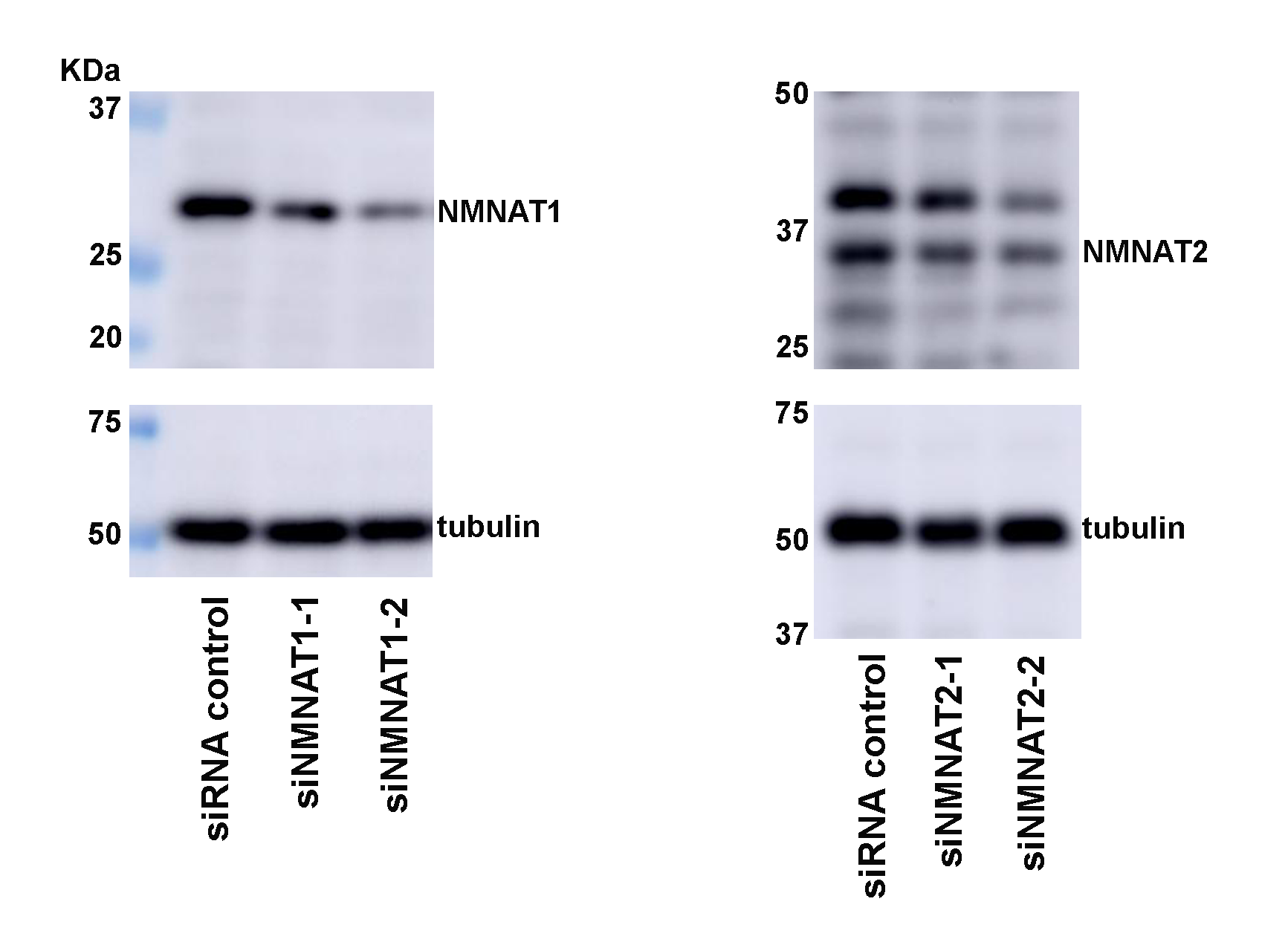


**Figure 3-figure supplement 3-source data 1**

Proteins are extracted from T98G cells transfected with siRNA and probed for NMNAT1 and NMNAT2. Tubulin was used as internal control.
