# Supplementary material for: NMNAT promotes glioma growth through regulating post-translational modifications of P53 to inhibit apoptosis
Source: eLife. 2021 Dec 17;10:e70046. doi: 10.7554/eLife.70046 (PMC8683086; doi:10.7554/eLife.70046)
Supplement: Figure 5—source data 1. [file elife-70046-fig5-data1.doc]

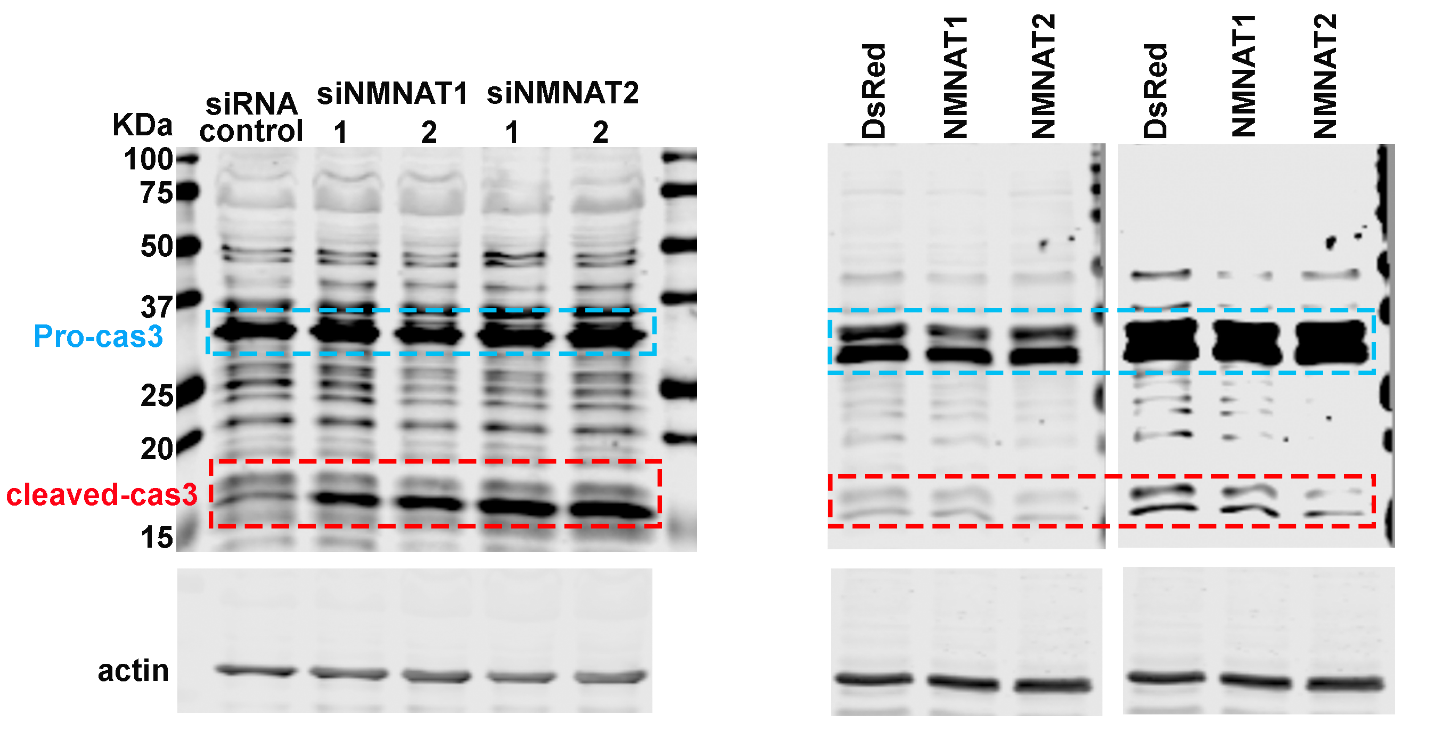


**Figure 5-source data 1**

The full blots for figure 5D and F. Proteins are extracted from T98G cells transfected with siRNA or plasmids and probed for Caspase-3 and β-actin. Pro-caspase3 and cleaved caspase3 bands are marked with blue dashed lines and red dashed lines separately. β-actin was used as internal control.
