# Supplementary material for: NMNAT promotes glioma growth through regulating post-translational modifications of P53 to inhibit apoptosis
Source: eLife. 2021 Dec 17;10:e70046. doi: 10.7554/eLife.70046 (PMC8683086; doi:10.7554/eLife.70046)
Supplement: Figure 5—figure supplement 2—source data 1. [file elife-70046-fig5-figsupp2-data1.doc]

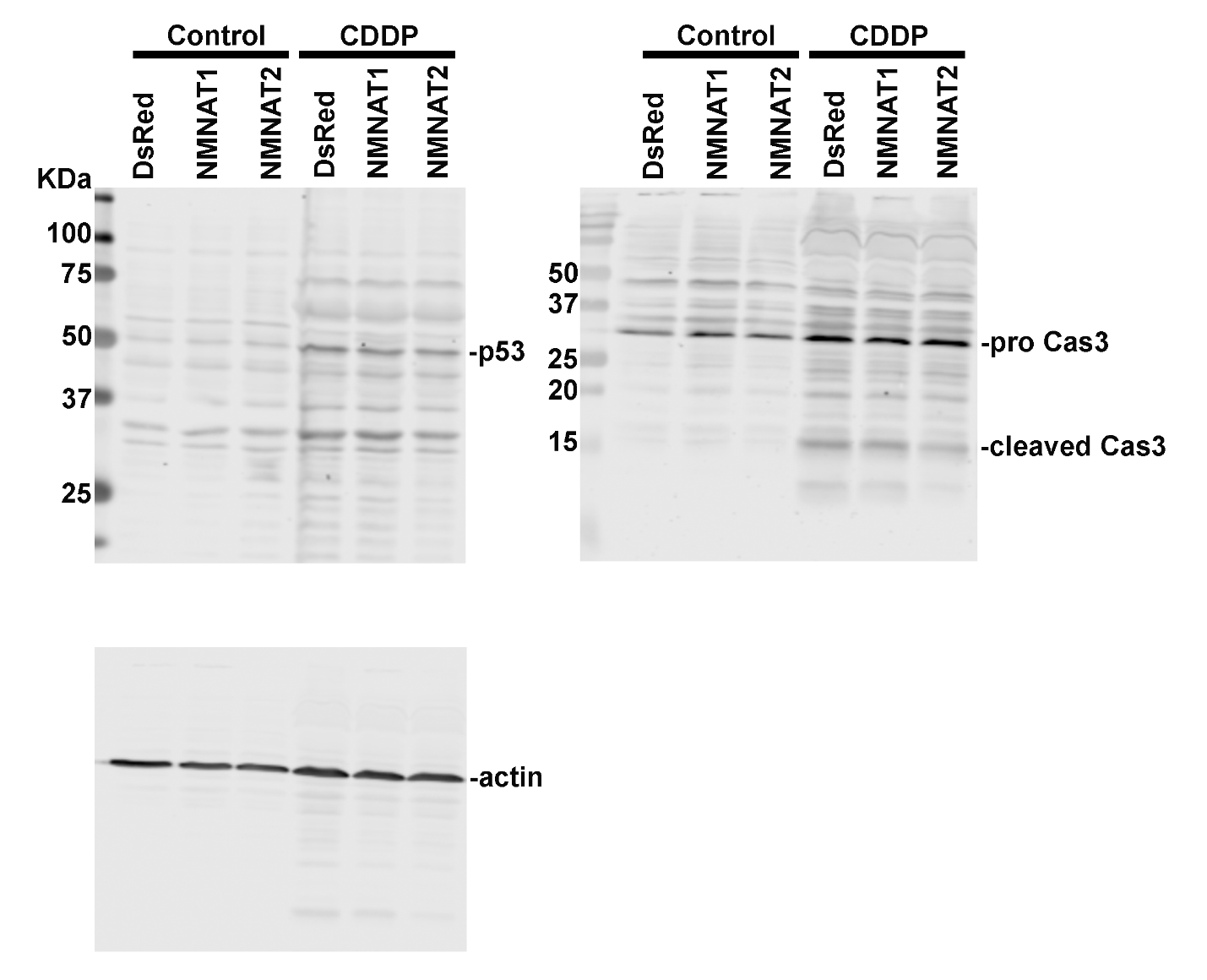


**Figure 5-figure supplement 2-source data 1**

U87MG cells were treated with CDDP and probed for p53, caspase-3 and β-actin. β-actin was used as internal control.
