# Supplementary material for: NMNAT promotes glioma growth through regulating post-translational modifications of P53 to inhibit apoptosis
Source: eLife. 2021 Dec 17;10:e70046. doi: 10.7554/eLife.70046 (PMC8683086; doi:10.7554/eLife.70046)
Supplement: Figure 8—source data 1. [file elife-70046-fig8-data1.doc]

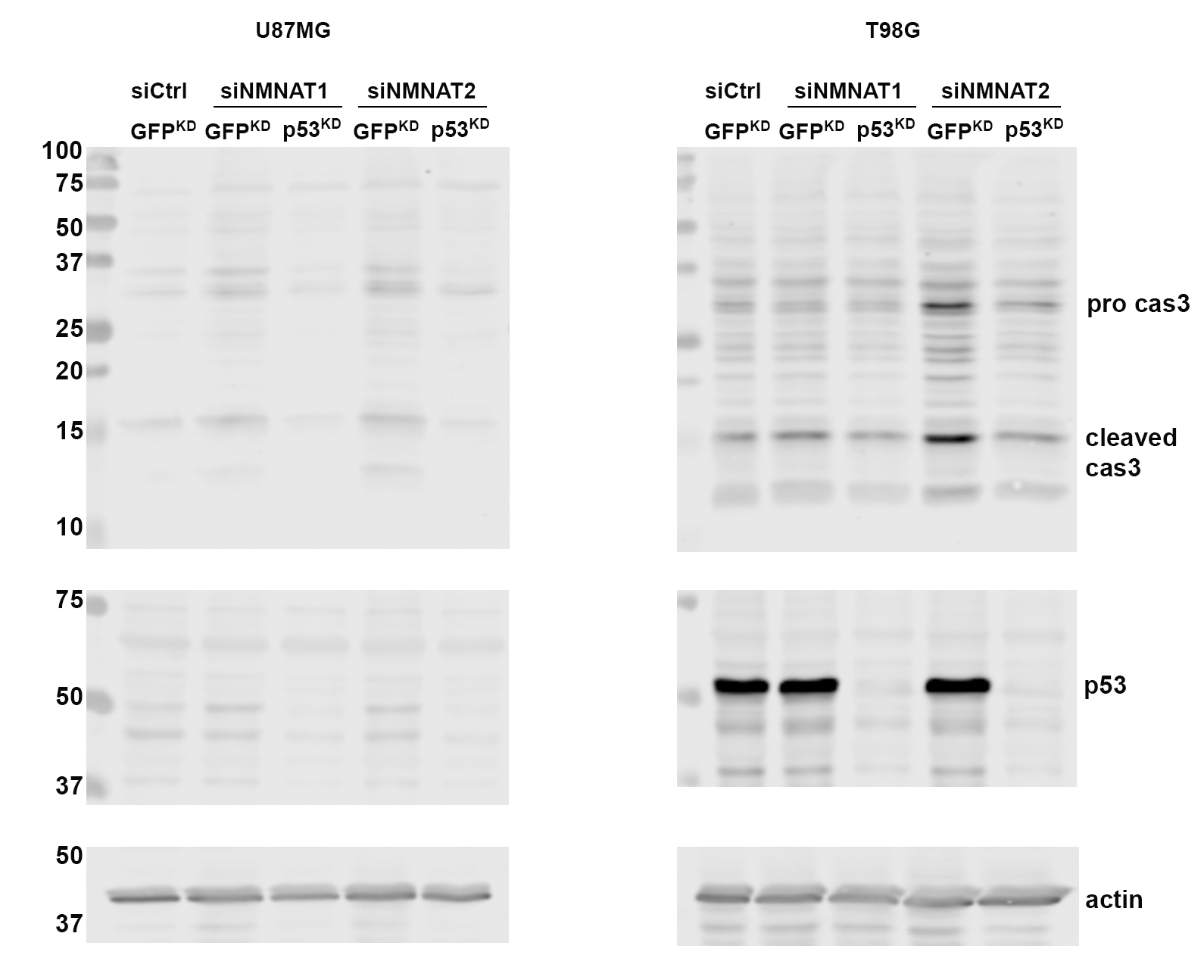


**Figure 8-source data 1**

The full blots for figure 8. Proteins are extracted from GFP or p53 knockdown U87MG and T98G cells transfected with siRNA and probed for Caspase-3, p53 and β-actin. β-actin was used as internal control.
