# Supplementary material for: NMNAT promotes glioma growth through regulating post-translational modifications of P53 to inhibit apoptosis
Source: eLife. 2021 Dec 17;10:e70046. doi: 10.7554/eLife.70046 (PMC8683086; doi:10.7554/eLife.70046)
Supplement: Figure 9—source data 1. [file elife-70046-fig9-data1.doc]

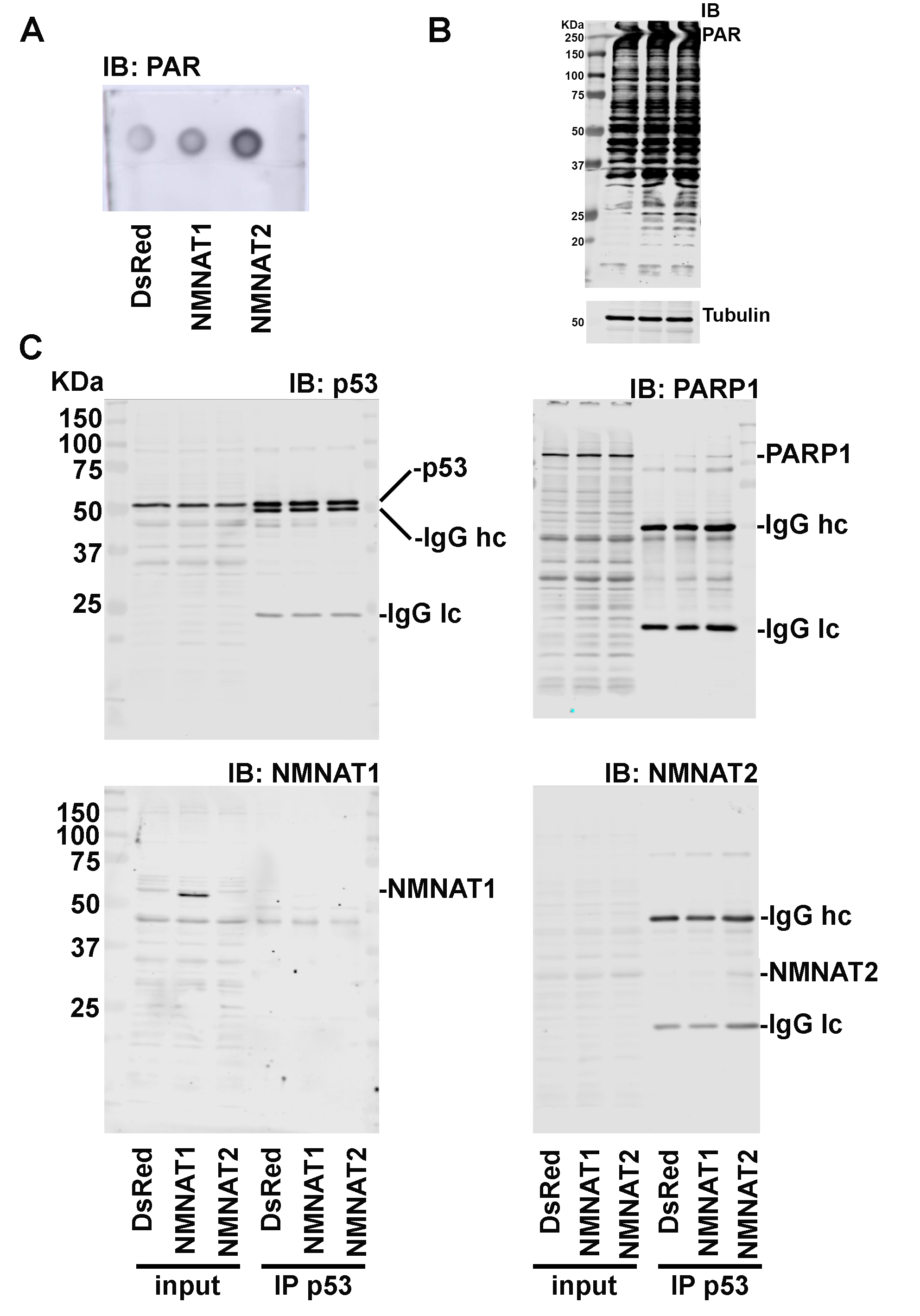


**Figure 9-source data 1**

The full blots for figure 8A, B and C. (**A, B**) Proteins were extracted from T98G cells transfected with plasmids for dot blot and western blot analysis using anti-PAR antibody. (**C**) Protein samples extracted from T98G cells transfected with DsRed, DsRed-NMNAT1 or NMNAT2 were immunoprecipitated (IP) with a p53 antibody and subjected to immunoblot (IB) analysis for p53, PARP1, NMNAT1 and NMNAT2.
