# Supplementary material for: NMNAT promotes glioma growth through regulating post-translational modifications of P53 to inhibit apoptosis
Source: eLife. 2021 Dec 17;10:e70046. doi: 10.7554/eLife.70046 (PMC8683086; doi:10.7554/eLife.70046)
Supplement: Figure 9—figure supplement 2—source data 1. [file elife-70046-fig9-figsupp2-data1.doc]

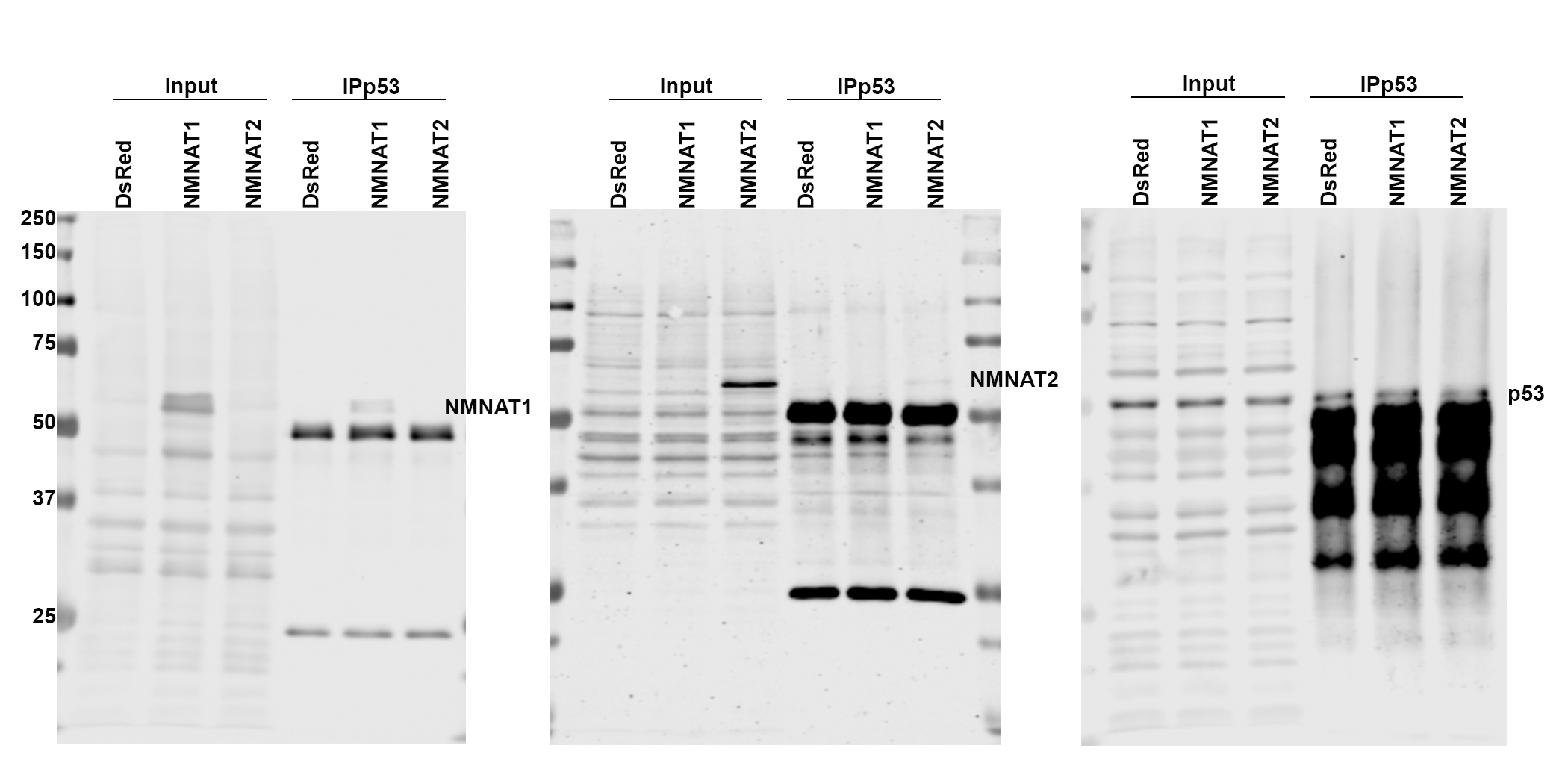


**Figure 9-figure supplement 2-source data 1**

The full blots for figure 9-figure supplement 2. Protein samples extracted from U87MG cells transfected with DsRed, DsRed-NMNAT1 or NMNAT2 were immunoprecipitated (IP) with a p53 antibody and subjected to immunoblot (IB) analysis for p53, NMNAT1 and NMNAT2.
