# Supplementary material for: NMNAT promotes glioma growth through regulating post-translational modifications of P53 to inhibit apoptosis
Source: eLife. 2021 Dec 17;10:e70046. doi: 10.7554/eLife.70046 (PMC8683086; doi:10.7554/eLife.70046)
Supplement: Figure 10—source data 1. [file elife-70046-fig10-data1.doc]

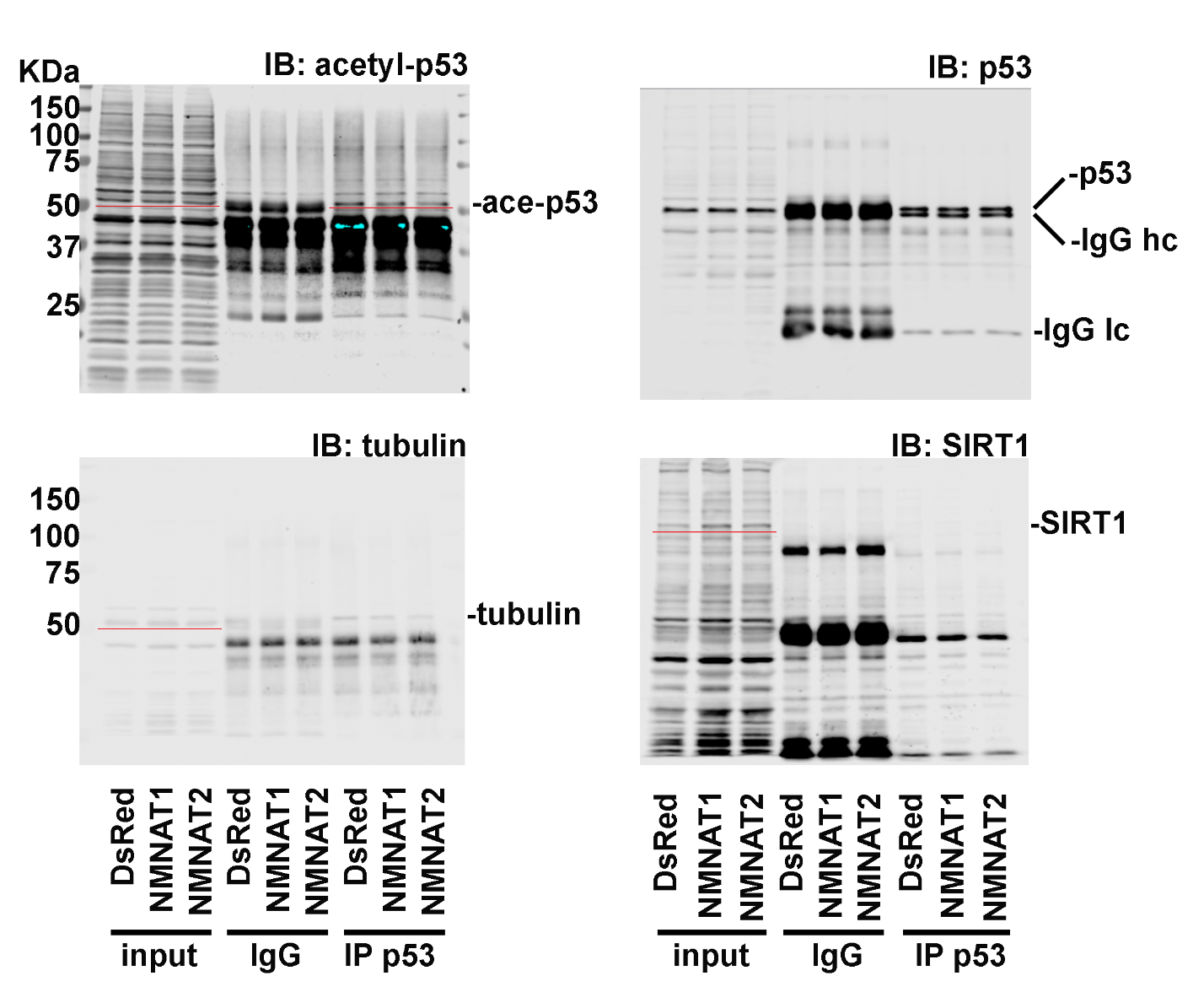


**Figure 10-source data 1**

The full blots for figure 9A. Protein samples extracted from T98G cells transfected with DsRed, DsRed-NMNAT1 or NMNAT2 were immunoprecipitated (IP) with a p53 antibody and probed for acetyl-p53 and SIRT1. Tubulin was used as internal control.
