# Supplementary material for: NMNAT promotes glioma growth through regulating post-translational modifications of P53 to inhibit apoptosis
Source: eLife. 2021 Dec 17;10:e70046. doi: 10.7554/eLife.70046 (PMC8683086; doi:10.7554/eLife.70046)
Supplement: Figure 10—source data 2. [file elife-70046-fig10-data2.doc]

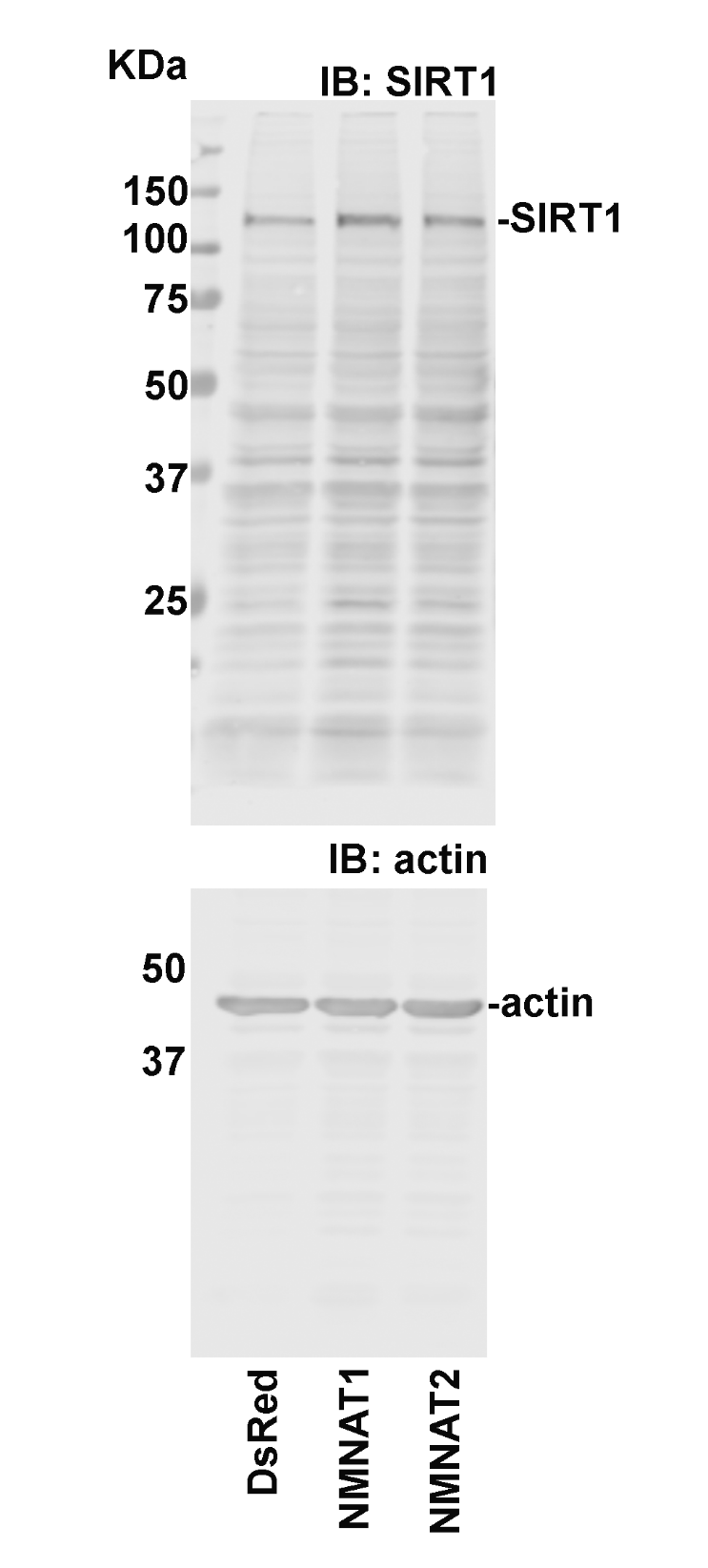


**Figure 10-source data 2**

The full blots for showing SIRT1 band with another more specific anti-SIRT1 antibody. Proteins were extract form T98G transfected with plasmids. β-actin was used as internal control.
